# Recently Top Trending Cancers in a Tertiary Cancer Hospital in Pakistan

**DOI:** 10.1007/s44229-023-00028-z

**Published:** 2023-03-16

**Authors:** Faisal Ali, Sadiq Hussain, Sajjad Ahmed Memon, Syed Shahid Iqbal

**Affiliations:** 1 Nuclear Institute of Medicine and Radiotherapy, Jamshoro, Pakistan; 2 Nuclear Medicine Oncology and Radiotherapy Institute, Nawabshah, Pakistan

**Keywords:** GLOBOCAN, Head and neck, Breast, Gynecological tumors, Esophagus, Lung, Colorectal, Liver, Lymphoma, Urinary tract, Prostate

## Abstract

Cancer is a leading cause of death, and its incidence is increasing, as reported in recent studies by GLOBOCAN. Cancer registry programs provide insights into currently trending tumors worldwide and aid in determining possible risk factors. This study was based on 7 years of cancer registry data recorded at NIMRA cancer hospital, Sindh, from 2015 to 2021. A total of 16,191 cancer patients were registered. In men, head and neck, lung, liver, colorectal and urinary tract cancers were most common. In women, breast cancer, head and neck cancer, gynecological tumors, esophageal cancer and colorectal cancer predominated. The overall data analysis indicated trending cancers in both sexes, including head and neck cancer (37.76%), breast cancer (13.83%), gynecological tumors (10.22%), esophageal cancer (5.18%), lung cancer (4.79%), colorectal cancer (4.27%), liver cancer (3.87%), lymphoma (3.16%), urinary tract cancer (3.11) and prostate cancer (1.53%). The mean age was 50.41 ± 11.78 years in men and 48.47 ± 11.88 years in women. Cancer prevalence has markedly increased worldwide, and is particularly alarming in developing countries. Various risk factors are involved in this increase, including the use of tobacco, areca nut, chewable tobacco, snuff or niswar. Current disease trends are substantially different from those in older studies at the institute.

## Introduction

Cancer is a leading cause of mortality worldwide among people of all ages [1]. Globally, large expenditures are allocated to fighting against this disease. According to published research, in 2000, 6 million deaths occurred, and 22 million people were living with this deadly disease. That study predicted that, on the basis of the rates at the time, 15 million new cases and approximately 10 million deaths would occur in 2020 [2]. In fact, approximately 19.3 million new cancer cases were registered, and approximately 10 million deaths were reported worldwide in 2020. According to global cancer incidence (GLOBOCAN), the global burden of cancer is expected to be 28.4 million by 2040, a number 47% higher than that in 2020 [3].

The rapid increases in cancer incidence rates have become a serious public health issue in Pakistan and a focus of continuing governmental efforts. Cancer is a multigenic and multicellular disease that can arise from all cell types [4]. In developing countries, chronic infections are responsible for causing more than 22% of cancers, and the use of betel nuts and tobacco remains the most important risk factor [5]. For future policymaking regarding cancer prevalence, cancer registries have been found to be a valuable source of information on associated cancer risk factors in genetic, epigenetic and epidemiological studies. Cancer demographic data captured in registries change over time, under the influence of cancer screening programs; the availability of cancer treatment and diagnostic facilities; and variations in genetic, epigenetic and environmental risk factors. Epidemiological studies improve understanding of changing trends in cancer risk factors, thus aiding in interpretation and forecasting of future cancer incidence [6–9]. Cancer is a non-communicable deadly disease that has recently been found to be the leading cause of death [10, 11]. Asian developing countries must take immediate steps to prevent and detect cancer.

## Materials and Methods

### Inclusion of Data

The NIMRA cancer hospital is one of the oldest cancer hospital facilities built by the Pakistan Atomic Energy Commission (PAEC); it is located in the South Sindh province of Pakistan. Its scope includes diagnosis, nuclear medicine, and treatment of malignant cancers with chemotherapy and radiotherapy. NIMRA cancer hospital is capable of performing research studies to examine current cancer trends and forecast future trends. The center is equipped with a well-established cancer registry software hospital management information system (HMIS) for data analysis. Data were registered from January 1, 2015 to December 31, 2021, and included a total of 161,191 patients with biopsies treated at NIMRA cancer hospital. Each patient was registered with an automatically generated unique ID in HMIS software. Data were categorized by year, sex and type of cancer for 7 years. The data were divided into tabulated and graphical distributions for analysis.

### Exclusion of Data

Miscellaneous diseases included pathologies present in low proportions and undefined primary cases, including melanoma, mesothelial, skin or soft tissue cases. Miscellaneous cases were excluded from the top ten trending cancer analyses.

## Results

Cancer registry statistics of the reported cases for a period of 7 years (2015–2021) and the percentage incidence in both sexes are shown in Table 1. Almost on average cancer incidence reported ratio is about 50% between both sexes annually.

**Table 1:** Registered cases from 2015 to 2021

Year	Male	Male (%)	Female	Female (%)	Total
2015	1139	51	1095	49	2234
2016	1058	52	977	48	2035
2017	1119	50	1119	50	2237
2018	1204	49	1253	51	2457
2019	1215	49	1264	51	2479
2020	1159	51	1113	49	2272
2021	1214	49	1263	51	2477
				Total	16,191

**Fig. 1:**
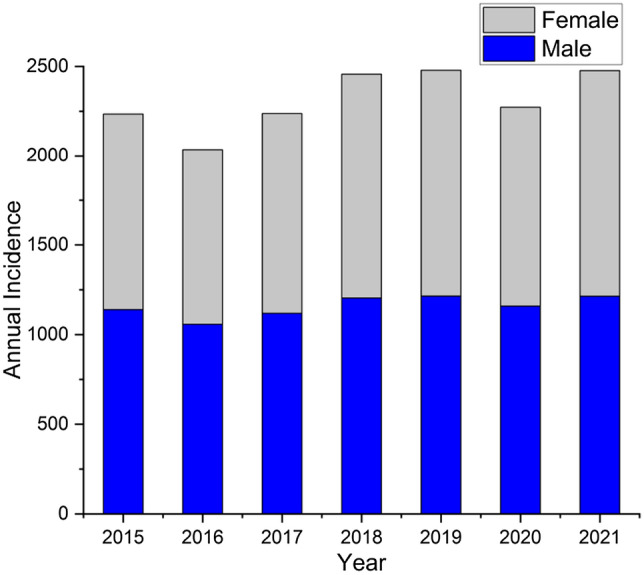
Graphical distribution of annually reported cases in both sexes

The graphical distribution of the reported cancer cases by year is shown in Fig. 1. The total number of registered cancer patients by year in Fig. 1 indicated the trends in reported cases over the past 4 years (2018–2021). The results indicated increased numbers of cancer patients per annum with respect to past data, except for the reported cancer cases in the year 2020 (because of the COVID-19 pandemic).

Reported cancer cases from 2015 to 2021 are categorized according to disease in Table 2. Cases in men and women were normalized to the most prevalent disease, which was predominantly head and neck cancer, in all annual data.

**Table 2:** Normalized proportions of annually registered cases from 2015 to 2021

Site	2015		2016		2017		2018		2019		2020		2021	
	*N*	Norm	*N*	Norm	*N*	Norm	*N*	Norm	*N*	Norm	*N*	Norm	*N*	Norm
Bone	34	0.05	32	0.043	50	0.06	32	0.03	28	0.03	29	0.03	26	0.03
Brain	36	0.05	28	0.037	31	0.04	40	0.04	39	0.04	17	0.02	27	0.03
Breast	312	0.42	239	0.316	267	0.34	350	0.37	372	0.37	329	0.35	370	0.39
Colorectal	96	0.13	80	0.106	96	0.12	92	0.1	92	0.09	113	0.12	123	0.13
Esophagus	71	0.09	85	0.112	115	0.15	128	0.14	144	0.14	154	0.16	143	0.15
Gallbladder	17	0.02	7	0.01	19	0.02	15	0.02	13	0.01	17	0.02	15	0.02
Head and neck	748	1	756	1	779	1	947	1	1008	1	939	1	937	1
Leukemia	32	0.04	23	0.03	4	0.01	7	0.01	4	0	5	0.01	8	0.01
Liver	92	0.12	91	0.121	104	0.13	103	0.11	78	0.08	65	0.07	91	0.1
Lung	138	0.18	147	0.194	120	0.15	119	0.13	96	0.1	81	0.09	75	0.08
Pancreas	9	0.01	12	0.016	13	0.02	11	0.01	6	0.01	12	0.01	8	0.01
Prostate	27	0.04	26	0.034	38	0.05	37	0.04	50	0.05	34	0.04	36	0.04
Stomach	22	0.03	27	0.035	24	0.03	18	0.02	38	0.04	25	0.03	37	0.04
Lymphoma	77	0.1	69	0.091	78	0.1	73	0.08	78	0.08	63	0.07	73	0.08
Urinary tract	83	0.11	63	0.084	82	0.11	66	0.07	60	0.06	62	0.07	86	0.09
Testicular tumors	36	0.05	25	0.033	30	0.04	26	0.03	37	0.04	35	0.04	33	0.04
Gynecological tumors	241	0.32	202	0.267	207	0.27	248	0.26	265	0.26	225	0.24	267	0.28
Miscellaneous	163	0.22	123	0.163	180	0.23	145	0.15	71	0.07	67	0.07	122	0.13

**Fig. 2:**
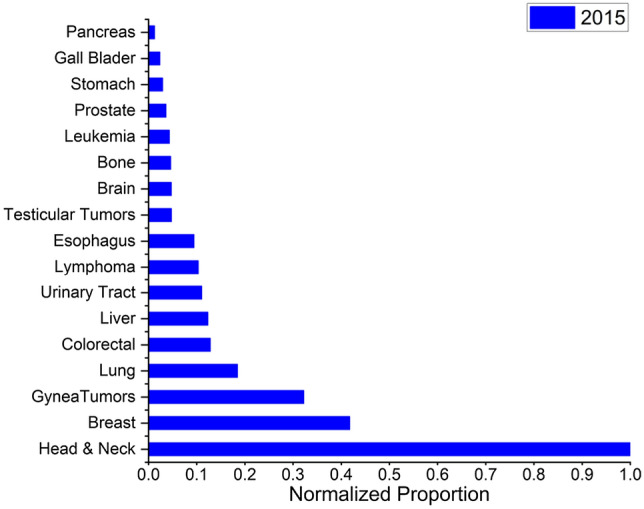
Graphical distribution of trending cancers registered in 2015

**Fig. 3:**
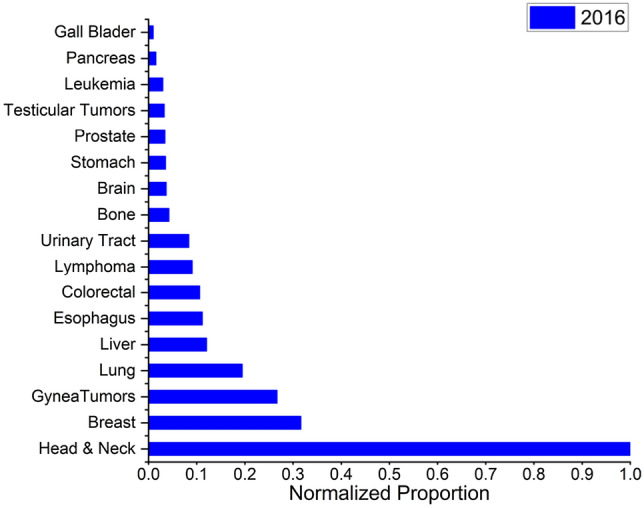
Graphical distribution of trending cancers registered in 2016

**Fig. 4:**
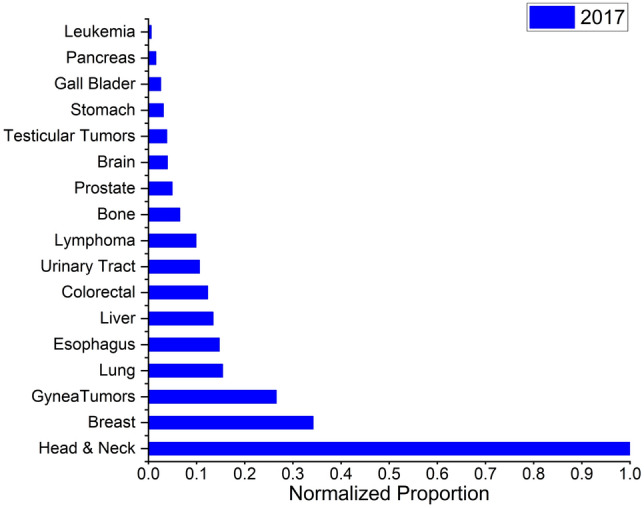
Graphical distribution of trending cancers registered in 2017

**Fig. 5:**
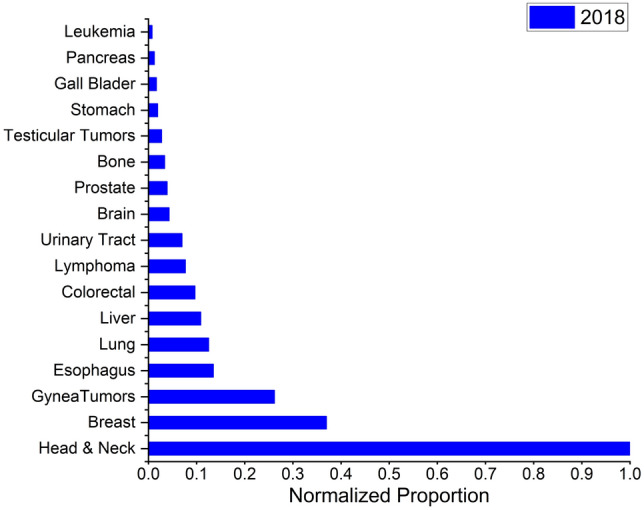
Graphical distribution of trending cancers registered in 2018

**Fig. 6:**
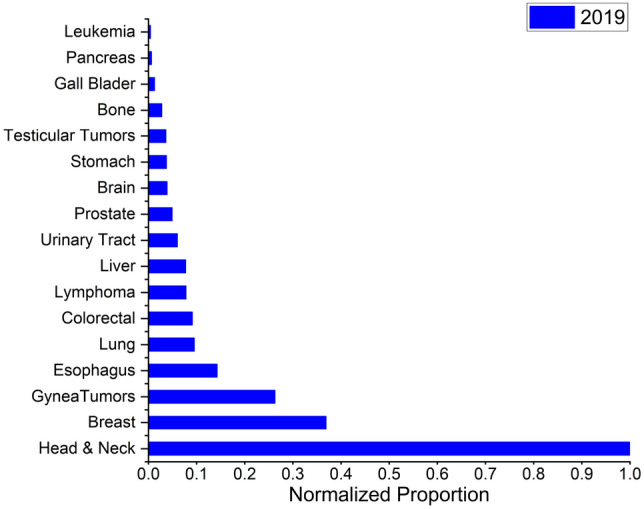
Graphical distribution of trending cancers registered in 2019

**Fig. 7:**
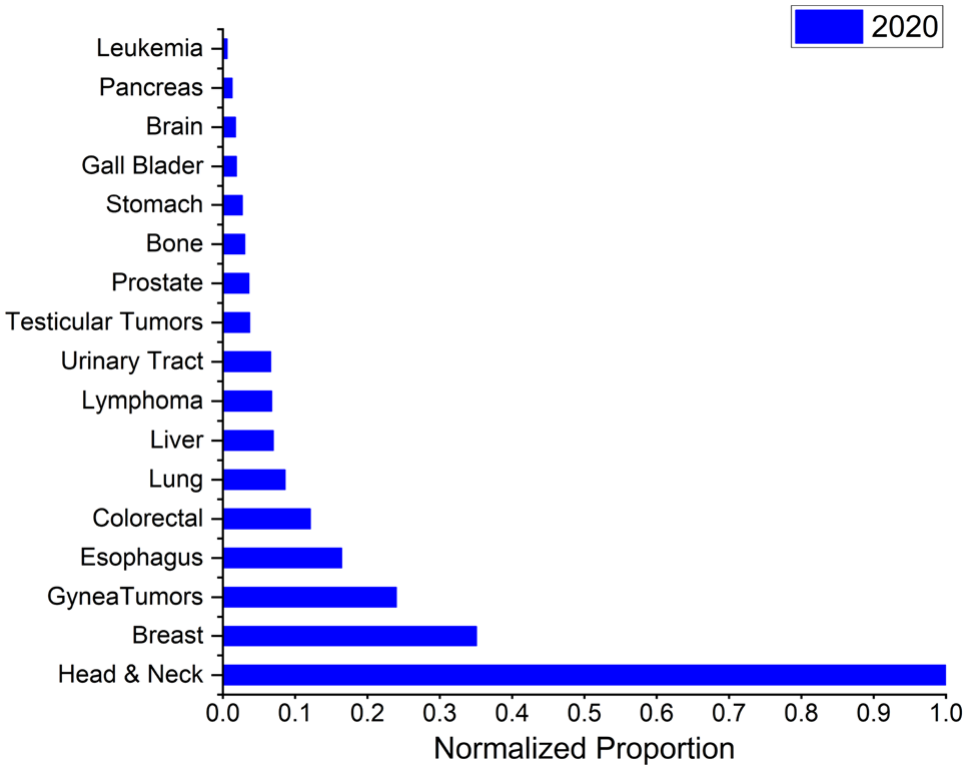
Graphical distribution of trending cancers registered in 2020

**Fig. 8:**
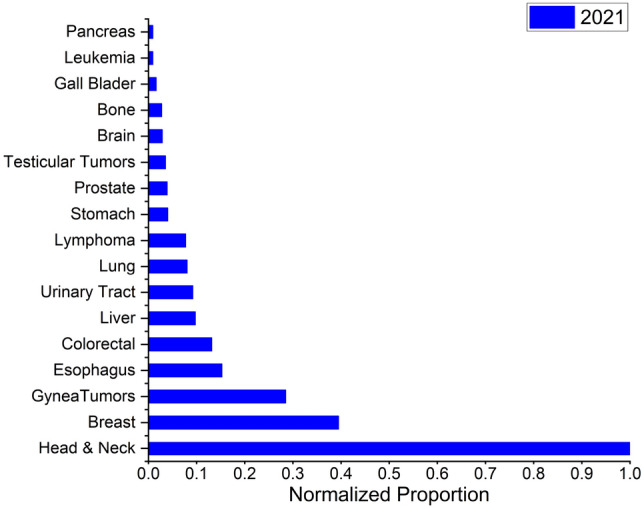
Graphical distribution of trending cancers registered in 2021

The normalized proportion graphs of incident disease are shown in Figs. 2, 3, 4, 5, 6, 7 and 8. Head and neck, breast and gynecological cancers had the most reported cases every year. Figure 2 shows that the top trending registered cancers in 2015 were head and neck cancer, breast cancer, gynecological tumors, lung cancer, colorectal cancer, liver cancer, urinary tract cancer, lymphoma, esophageal cancer and testicular tumors. Figure 3 shows that the top trending registered cancers in 2016 were head and neck cancer, breast cancer, gynecological tumors, lung cancer, liver cancer, esophageal cancer, colorectal cancer, lymphoma, urinary tract cancer and bone cancer. Figure 4 shows that the top trending registered cancers in 2017 were head and neck cancer, breast cancer, gynecological tumors, lung cancer, esophageal cancer, liver cancer, colorectal cancer, urinary tract cancer, lymphoma and bone cancer. Figure 5 shows that the top trending registered cancers in 2018 were head and neck cancer, breast cancer, gynecological tumors, esophageal cancer, lung cancer, liver cancer, colorectal cancer, lymphoma, urinary tract cancer and brain cancer. Figure 6 shows that the top trending registered cases in 2019 were head and neck cancer, breast cancer, gynecological tumors, esophageal cancer, lung cancer, colorectal cancer, lymphoma, liver cancer, urinary tract cancer and prostate cancer. Figure 7 shows that the top trending registered cases in 2020 were head and neck cancer, breast cancer, gynecological tumors, esophageal cancer, colorectal cancer, lung cancer, liver cancer, lymphoma, urinary tract cancer and testicular tumors. Figure 8 shows that the top trending registered cases in 2021 were head and neck cancer, breast cancer, gynecological tumors, esophageal cancer, colorectal cancer, liver cancer, urinary tract cancer, lung cancer, lymphoma and stomach cancer.

Among 16,191 registered cancer cases, 8107 were in men, and 8084 were in women. Table 3 depicts the overall distribution of 16,191 reported cases over the 7 years by sex. The aggregated cases in both sexes included 6113 (37.76%) head and neck cancer cases, 2239 (13.83%) breast cancer cases, 1654 (10.22%) gynecological tumor cases, 838 (5.18%) esophageal cancer cases, 776 (4.79%) lung cancer cases, 692 (4.27%) colorectal cancer cases, 626 (3.83%) liver cancer cases, 511 (3.16%) lymphoma cases, 503 (3.11%) urinary tract cancer cases, 248 (1.53%) prostate cancer cases, 232 (1.43%) bone cancer cases, 221 (1.36%) testicular tumor cases, 217 (1.34%) brain cancer cases, 191 (1.18%) stomach cancer cases, 106 (0.65%) gallbladder cancer cases, 85 (0.52%) leukemias, and 72 (0.44%) pancreatic cancer cases.

**Table 3:** Classification of annual reported cases from 2015 to 2021

Disease	Male	Male (%)	Female	Female (%)	*N*	*N* (%)
Head and neck	3970	24.52	2143	13.24	6113	37.76
Breast	33	0.20	2206	13.62	2239	13.83
Gynecological tumors	0	0.00	1654	10.22	1654	10.22
Esophagus	317	1.96	521	3.22	838	5.18
Lung	617	3.81	159	0.98	776	4.79
Colorectal	423	2.61	269	1.66	692	4.27
Liver	499	3.09	127	0.78	626	3.87
Lymphoma	328	2.03	183	1.13	511	3.16
Urinary tract	356	2.20	147	0.91	503	3.11
Prostate	248	1.53	0	0.00	248	1.53
Bone	148	0.91	84	0.52	232	1.43
Testicular tumors	221	1.36	0	0.00	221	1.36
Brain	143	0.88	74	0.46	217	1.34
Stomach	117	0.72	74	0.46	191	1.18
Gallbladder	30	0.19	76	0.47	106	0.65
Leukemia	54	0.33	31	0.19	85	0.52
Pancreas	46	0.28	26	0.16	72	0.44
Miscellaneous	557	3.44	310	1.91	867	5.35
**Total**					**16,191**	

**Fig. 9:**
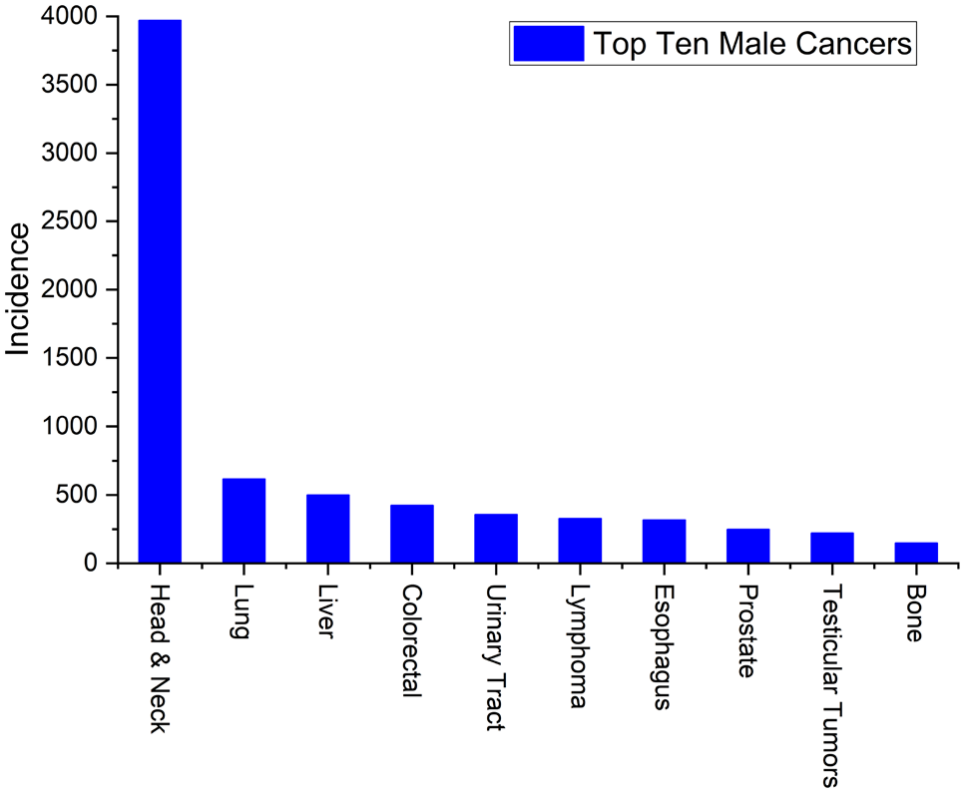
Top ten trending cancers in men

In men, the top trending cancers included head and neck cancer, lung cancer, liver cancer, colorectal cancer, urinary tract cancer, lymphoma, esophageal cancer, prostate cancer, testicular tumors and bone cancer, as shown in Fig. 9.

**Fig. 10:**
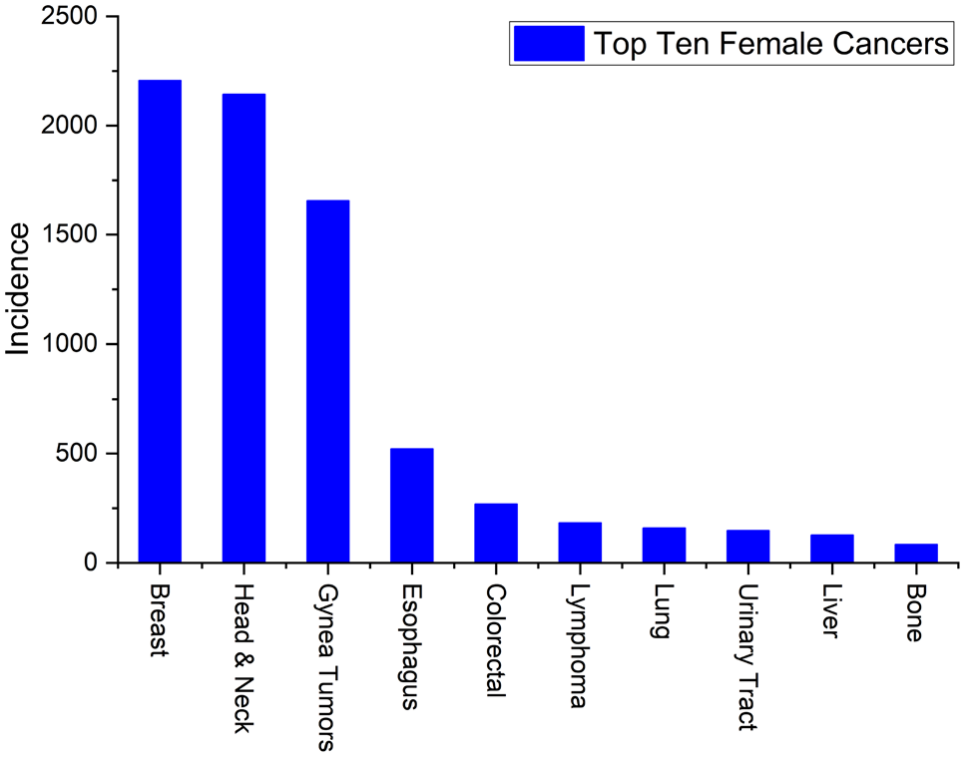
Top ten trending cancers in women

In women, the top trending cancers included breast cancer, head and neck cancer, gynecological tumors, esophageal cancer, colorectal cancer, lymphoma, lung cancer, urinary tract cancer, liver cancer and bone tumors, as shown in Fig. 10.

**Fig. 11:**
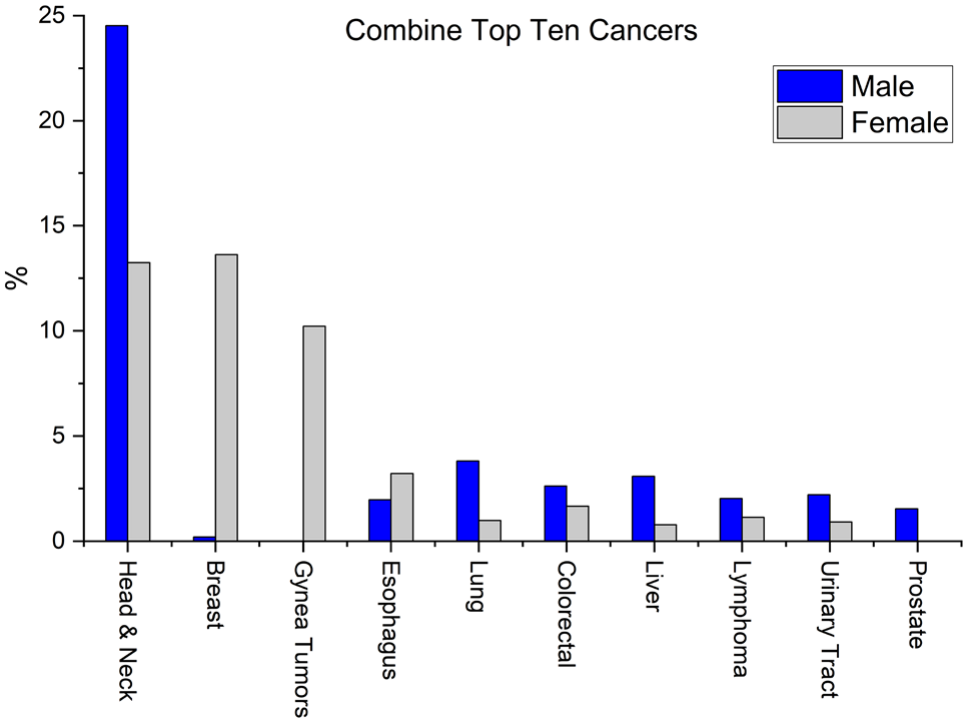
Top ten trending cancers in both sexes

An overall analysis of the data indicated that the top trending cancers in men and women included head and neck cancer, breast cancer, gynecological tumors, esophageal cancer, lung cancer, colorectal cancer, liver cancer, lymphoma, urinary tract cancer and prostate cancer, as shown in Fig. 11.

## Discussion

The World Bank categorizes Pakistan as a low-middle-income country. Head and neck cancers are among the top ten cancers globally and are the top trending cancer reported in Pakistan. No national cancer registry data were available for comparison, and this was an institution-based study. Trending cancer data from our center indicated that the cancer incidence in both sexes was approximately 50%, and the predominant reported disease was head and neck cancer. Among 16,191 reported cases, the overall burden of head and neck cancers was 37.38%: 24.52% in men and 13.24% in women. The main factors contributing to these trends were the use of tobacco, areca nut, chewable tobacco, snuff, niswar, etc. [12–15]. Areca nut is used in numerous scented forms, and betel quid (including betel leaf, areca nut, sweeteners, condiments, slaked lime, and tobacco) is used in most areas of Pakistan and is the major source of carcinogenesis for head and neck cancers [16]. Various cancers are included in the head and neck region, whereas oral cancers are the most common squamous cell carcinomas. In developing countries, compared with the Western world, squamous cell carcinomas differ in disease sites, molecular biology and risk factors. In developing countries, owing to financial limitations, poverty, illiteracy and access to appropriate health care centers are the major barriers to diagnosis and treatment; consequently advanced stage presentation of the disease occurs in most cases [17–19].

Breast cancer is diagnosed in Pakistan a decade earlier than in Western countries [20]. From the overall data analysis, breast cancer is the second most common cancer overall and is the first most common cancer in women in Pakistan [21]. In this study, the breast cancer incidence was 13.62% in women. Breast cancer is heterogeneous in nature, and its subtypes include luminal A, luminal B, HER2 and basal-like triple-negative [22]. Reproductive factors and weight gain are associated with luminal A and luminal B [23].

Menarche at an early age, menopause at a late age, and full-term pregnancy at a late age are associated with a modest range of risk of breast cancer development. Enhanced use of estrogen and progestogen has been linked with early menarche, late menopause, and a shortened menstrual cycle. In addition, the use of oral contraceptives aggravates the risk factors of breast cancer [24]. The breast cancer prevalence is 2.5 times higher in Pakistan than in its neighboring countries. Much research remains necessary to establish cultural, socioeconomic, religious, psychosocial and psychological factors [25].

Gynecological malignancies were the third most common disease in the overall reported data analysis, at 10.22%. Numerous risk factors are associated with these malignancies, e.g., financial instability, compromised life (living in slums, early age menarche, treatment of infertility, use of oral contraceptives, etc. Financial limitations, and a lack of education and health awareness often cause patients to visit spiritual healers rather than physicians [26].

Esophageal cancer is an aggressive lethal disease with a high mortality rate. Worldwide, approximately 90% of biopsied cancers are classified as squamous cell cancer and adenocarcinoma [27]. Epidemiology studies can help understand the history and risk factors, thus aiding in early diagnosis and treatment of esophageal cancers [28]. In our study, the incidence of esophageal cancers was low, at 1.96%, in men but was 3.22% in women. The overall esophageal cancer burden recorded in this study was 5.18%. Esophageal malignancies have alarmingly increased in women with respect to previously reported values. Esophageal cancer shows geographic variation internationally, and high and increasing rates have been observed in some areas of Asia. Environmental factors, dietary habits (nutritional factors) and the use of tobacco are the major factors associated with the development of esophageal cancer, whereas rich diets in vegetables and fresh fruits can decrease the risk [29, 30].

Lung cancer is a common malignant neoplasm worldwide in both sexes. The overall burden of lung cancer in this study was 4.79%: 3.81% in men and 0.98% in women. The main etiological risk factor for lung malignancies is the use of tobacco. Other factors associated with lung cancers are genetic susceptibility, inappropriate diet, air pollution and occupational radiation exposure. Lung cancer is a highly preventable malignancy and remains the most common and deadly disease globally. Comprehensive research addressing all possible risk factors in the development of lung diseases must be performed to support future prevention and management efforts [31, 32].

In the Western world, colorectal malignancies are the fourth most common cancer. The trends in reported colorectal cancers in this study indicated that the overall burden of the disease was 4.27%: 2.61% in men and 1.66% in women. Possible risk factors associated with colorectal cancers include lifestyle; habits; age; chronic disease history; and mutations targeting oncogenes, genes associated with DNA repair mechanisms and tumor suppressor genes [33–35].

Liver malignancies, e.g., hepatocellular carcinoma (HCC), are the second leading cause of death globally. In Pakistan, HCC in men markedly increased in the past two decades and might become the most common malignancy in the future. The overall burden of liver malignancy was found to be 3.87%: 3.08% in men and 0.78% in women. Possible risk factors for lung malignancies are hepatitis-B and hepatitis-C, whereas in some regions in developing countries, the disease burden due to hepatitis-B has decreased because of vaccination. A substantial number of the cases reported in tertiary care hospitals treating hepatocellular diseases are hepatitis-C positive, and hepatitis-C is a well-established risk factor for liver carcinogenesis. A lack of awareness of liver diseases among the general public has substantially increased the incidence rate in Pakistan with respect worldwide incidence. The cancer registry database and screening framework allowed us to determine possible risk factors, and support prevention and education efforts for hepatitis and HCC. Owing to the lack of availability of health education and dedicated facilities, patients with liver diseases usually present in advanced stages and have no definitive treatment options [36, 37].

The overall burden of lymphomas was 3.16% in both sexes. Certain autoimmune and chronic inflammatory conditions, such as the immune system disorder Sjogren's syndrome and rheumatoid arthritis, have consistently been associated with an enhanced risk of malignant lymphomas [38]. In regions with endemic parasites, cancer is also associated with parasitic infection.

Urinary tract infections include the urinary system, bladder, urethra, ureters and kidneys [39]. The reported cases of urinary tract malignancies were 3.11% in both sexes: 2.20% in men and 0.91% in women. Urinary tract cancers range from small benign lesions to malignant neoplasms. Bladder malignancy is found predominantly in urinary tract cancers. Delayed diagnosis is associated with poor prognosis in the treatment of urinary tract malignancies. Early detection and follow-up pose major challenges in the management of the disease [40].

Prostate cancers have continually increased in recent years. Our overall data analysis indicated a 1.53% burden of prostate cancer. The causes of prostate cancers are more poorly understood than those of other tumors. Researchers continue to identify risk factors for prostate cancers. The known risk factors include familial and genetic factors. Understanding predictors will enable better control and prevention of the disease [41].

The top ten trending cancers accounted for 87.70% of the 16,191 total reported cases, whereas low proportion disease and miscellaneous tumors accounted for 12.30%.

## Conclusion

Cancer is a leading cause of death globally, and its rate of incidence is increasing. Cancer trends differ between developing countries and the Western world. In this study, a substantial number of reported cases were head and neck (oral cavity) cancer, breast cancer, gynecological cancer, esophageal cancer, lung cancer, colorectal cancer and liver cancer. Various risk factors are involved in the increase in this deadly disease, e.g., use of tobacco, areca nut, chewable tobacco, snuff, niswar, etc. [12–15]. The observed disease trends have substantially changed with respect to older studies from our institute [42].

## Data Availability

The raw data used in this study may be requested from the authors.

## Abbreviations

GLOBOCAN: Global cancer incidence
NIMRA: Nuclear Institute of Medicine and Radiotherapy
PAEC: Pakistan Atomic Energy Commission
HMIS: Hospital Management and Information System
HER2: Human epidermal growth factor receptor 2
HCC: Hepatocellular carcinoma
